# Antioxidant Activity and Phenolic Profile of Selected Organic and Conventional Honeys from Poland

**DOI:** 10.3390/antiox9010044

**Published:** 2020-01-04

**Authors:** Michał Halagarda, Sabrina Groth, Stanisław Popek, Sascha Rohn, Vasilisa Pedan

**Affiliations:** 1 Department of Food Product Quality, Cracow University of Economics, Sienkiewicza 5, 30-033 Kraków, Poland; michal.halagarda@uek.krakow.pl (M.H.); popeks@uek.krakow.pl (S.P.); 2 Hamburg School of Food Science, Institute of Food Chemistry, University of Hamburg, Grindelallee 117, 20146 Hamburg, Germany; sabrina.groth@chemie.uni-hamburg.de (S.G.); rohn@chemie.uni-hamburg.de (S.R.); 3 Life Sciences and Facility Management, Zurich University of Applied Sciences, 8820 Wädenswil, Switzerland

**Keywords:** honey, polyphenols, pollen analysis, antioxidants

## Abstract

Honey is a natural food product hypothesized to have significant health-beneficial value. The results of recent studies indicate that the biological activity of honey can also be ascribed to phenolic compounds and their antioxidant activity. The aims of this study were: To determine the phenolic profiles of several varieties of Polish honey and their correlation with various factors influencing the quality of honey, plus to verify the impact of production method (organic/conventional) and the pollen content on these profiles. In total, 11 organic and 11 conventional honey samples from Poland were investigated. The botanical origin of the samples was identified through melissopalynological analysis, whereas individual phenolic compounds were determined by the LC/MS analysis. The Folin–Ciocalteau assay was used for the determination of the total phenolic content (TPC). Moreover, the CIE L*a*b* color values were measured and matched with the above-mentioned parameters. The results of the study contribute to the discussion on the health benefits of organic farming. It was found that chrysin may act as a potential indicator compound. The study confirms the existence of the link between TPC and color, and it shows that there is a correlation between pinocembrin and galangin, two compounds that are reported to ameliorate insulin resistance.

## 1. Introduction

Honey, as a natural food product, is believed to provide significant nutritional, as well as preventive and curative, value. Numerous literature sources point out that regular consumption of honey is one of the best forms of providing the organism with easily digestible carbohydrates, bioactive and bacteriostatic substances, organic acids, amino acids, as well as certain macro- and micronutrients [1]. As honey originates from various single and/or multiple plant species, it is also a natural source of antioxidants such as phenolic acids, carotenoids, proteins, certain enzymes and to some extent Maillard reaction products [2,3,4,5,6]. However, the contents and composition of antioxidant compounds depend to a large extent on the floral source, and thus also on the variety of honey [7,8]. Moreover, the composition of honey is influenced by various ecophysiological factors of the field (such as global radiation, temperature, soil conditions) and processing conditions (temperature in the hive or during the preparation of the final honey) [9].

Recent studies by Alvarez-Suarez et al. [10] have shown that the biological activity of honey can be associated with the content of phenolic compounds found in it. Due to their chemical structure, phenolic compounds are the most effective antioxidants in the context of food ingredients [2,11], but they also provide anti-inflammatory, anti-proliferative, and antimicrobial activity [10]. Consequently, research suggests that phenolic compounds are also mainly responsible for the antioxidant activity of honey [8,12]. 

As shown by Badolato et al. [13], the basic composition of phenolic compounds in different varieties of honey is quite similar and includes *p*-coumaric acid, eugenol, ferulic acid, caffeic acid, pinobanksin, pinocembrin, chrysin, quercetin, apigenin, and naringin in different proportions. Nonetheless, honey can contain some variety-specific compounds that may be used as markers of their botanical origin [3,14]. Hence, the floral source, along with the geographical origin and climatic conditions, influence antioxidant properties of honey [15,16,17,18]. Consequently, the properties of the same variety of honey might be different in the diverse regions of the world.

As mentioned before, the literature suggests the dependence of the total phenolic content on the antioxidant activity of honey [19,20,21], but it may also be responsible for its color, e.g., [22,23,24]. However, in Polish honeys, only a few studies related to the content of phenolic compounds, color, antioxidant activity, etc., have been conducted.

When looking at the quality of honey, the production method plays an interesting role as well. However, because the results of the studies investigating the influence of organic farming on the phenolic compounds in vegetables and fruits are still not comprehensive enough and more or less inconclusive [25], this issue needs further analysis, especially considering transformation products such as honey, where plant extracts and pollen are found. The results obtained so far suggest that there is still a need to deepen the research on antioxidant properties of honey, especially considering the influence of pollens contained in it [22,23,26,27,28,29].

Considering the above-mentioned issues, the main aim of this study was to determine the phenolic profile of Polish honeys with a special focus on the influence of production method (organic vs. conventional) and the pollen content on the concentrations of phenolic compounds. Moreover, the study aimed at the recognition of possible correlations between the contents of specific phenolic compounds, color parameters, total phenolic content, and antioxidant activity of the samples tested.

## 2. Materials and Methods 

### 2.1. Reagents

The following phenolic compounds were bought from Sigma-Aldrich Chemie GmbH (Buchs, Switzerland): Apigenin, chrysin, caffeic acid, *p*-coumaric acid, ferulic acid, galangin, luteolin, pinocembrin, quercetin, and kaempferol. Acetonitrile, methanol, water, and formic acid (LC-MS grade) were also purchased from Sigma-Aldrich. The Folin-Ciocalteau reagent (2 M) and anhydrous sodium carbonate (Sigma-Aldrich,) were used to measure the total phenolic content. To measure the antioxidant activity, 2,2-diphenyl-1-picrylhydrazyl (DPPH; Sigma-Aldrich) was used.

### 2.2. Samples

The study comprised 22 honey samples of 11 varieties, which were directly specified by the beekeepers, considering the location of the hive, the season, and all available floral sources. Each variety was represented by an organic and a conventional sample. The samples came from the following regions of Poland: Podlaskie, (samples Nos. 1, 4, 12, 14, 21, 22), Podkarpackie (samples Nos. 2, 3, 5, 6), Pomorskie (samples Nos. 7, 10, 17, 19), Mazowieckie (samples No. 9, 16, 18), Zachodniopomorskie (sample No. 11), Lubelskie (samples Nos. 13, 15), Świętokrzyskie (sample No. 20), Lubuskie (sample No. 8). They were bought directly the apiaries and in organic food stores.

The botanical origin of the honey samples was determined by the melissopalynological analysis, as established by the *International Commission of Bee Botany* [30]. In the case of the samples of honeydew honey, specific electrical conductivity measurements of aqueous 20% solutions were made with the use of a CX-721 sensor (ELMETRON, Zabrze, Poland). The detailed characteristics of the honey samples, along with pollen analysis and conductivity test results, are presented in Table 1.

The results of melissopalynological analyses are shown as the percentage of each pollen type content. According to Flores et al. [31], pollens are classified as dominant (concentration equal to or upper than 45%), accompanying (concentration between 15% and 45%), or important (concentration between 3% and 15%). Moar [32] indicated that the content of 45% is a universal and minimal limit for the classification of honey as monofloral. As the results obtained showed that in most of the cases the classification of honeys made by their producers was incorrect, the research outcomes were analyzed and discussed with consideration of the determined pollen concentrations and conductivity.

### 2.3. Sample Preparation 

In the present study, 2 g honey samples were weighed into a 50 mL centrifuge vessel and mixed with 2.5 mL distilled water, adjusted to pH 2.0 with concentrated HCl, and homogenized for 10 min. The extraction of phenolic compounds and the enrichment using Amberlite XAD-2 resin were performed according to Yao et al. [33]. The stationary phase was prepared by soaking 7 g of the Amberlite XAD-2 resin in distilled water for 10 min. Further, the fluid honey sample was mixed with 7 g Amberlite XAD-2 for 30 min. The Amberlite particles were then packed in a 6 mL glass column (Macherey-Nagel GmbH & Co. KG, Oensingen, Switzerland) and washed with 10 mL acidified water with the addition of 10 mL distilled water. The Amberlite-resin-bound phenolic compounds were eluted subsequently with 7 mL methanol and 7 mL acetonitrile. The eluent was dried overnight under nitrogen at 20 °C with a sample concentrator (Portmann Instruments AG, Biel-Benken, Switzerland). For the RP-HPLC-ESI/MS analysis, the sample was subsequently dissolved in 200 µL methanol and 200 µL acetonitrile and stored at −20 °C. Each sample was prepared in triplicate, and each one was analyzed three times over (mean ± SD). 

### 2.4. Characterization of Chemical Components Using Folin–Ciocalteau Assay

The advantage of the photometrical Folin–Ciocalteau assay is given by a quick analysis from a crude matrix extract. The TPC of each honey extract was determined according to Singleton and Rossi [34] with small modifications, as described by Pedan et al. [35].

### 2.5. Quantification of Chemical Components Using LC-DAD/ESI-MSD

The identification of the individual compounds in the honey extracts was confirmed by RP-LC-DAD/ESI-MSD. This was performed on an Agilent 1200 series liquid chromatography and quadrupole mass spectrometer with electrospray ionization interface (LC-MSD 6120, G6100 series, Agilent Technologies AG, Waldbronn, Germany). The honey extracts were analyzed using a gradient mixture of water-formic acid (99.9:0.1, v:v) (solvent A) and acetonitrile-water-formic acid (94.9:5:0.1, v:v:v) (solvent B). A 3.0 × 150 mm Eclipse XDB-C18 (3.5 µm) column (Agilent Technologies AG) was used. The separation was effected using a linear gradient at 38 °C with a flow of 0.25 mL/min as follows: 3% B at 0–5 min, 3–6% B at 5–8 min, 6–11% B at 8–20 min, 11–12% B at 20–25 min, 12–17% B at 25–32 min, 17–20% B at 32–38 min, 20–28% B at 38–44 min, 28–31% B at 44–47 min, 31–38% B at 47–51 min, 38–45% at 51–54 min, 45–50% at 54–58 min, 50–90% at 58–61 min, 90% B at 61–63 min and 90–1% B at 63–64 min.

### 2.6. RP-HPLC-Online-TEAC

The analysis of methanolic honey extracts was performed using an HPLC system (S5050 Knauer GmbH, Berlin, Germany) equipped with a Knauer S1000 quaternary solvent manager pump and Knauer S3950 autosampler. A Knauer S2600 diode array detector (set at 280, 325, 350, 365 nm) was used for detection of the positive peaks, whereas a Knauer S2550 UV-VIS detector (set at 414, 734 nm) was used for detection of the negative peaks. The system was controlled with ClarityChrom 3.0.7.662 software (Knauer GmbH). The separation was carried out on a Luna^®^ 5 µm phenyl-hexyl 100 Å (250 × 4.60 mm; Phenomenex Inc., Aschaffenburg, Germany) column at a temperature of 21 °C and a flow rate of 0.7 mL/min. The injection volume was 30 µL. A binary gradient system measurement was performed with the eluent water-formic acid (99.9:0.1, v:v) (solvent A) and acetonitrile-formic acid (99.9:0.1, v:v) (solvent B) for the methanolic extracts: 10% B isocratic at 0–5 min, 10–15% B at 5–10 min, 15% B isocratic at 10–30 min, 15–20% B at 30–40 min, 20–55% B at 40–60 min, 55–95% B at 60–65 min, 95% B isocratic at 65–70 min, 95–10% B at 70–75 min, and 10% B isocratic at 75–85 min.

For the purpose of the online-TEAC assay, an ABTS·^+^ solution (100 µM) was added at a flow rate of 0.7 mL/min using an auxiliary Knauer S100 pump with a T-valve. The scavenging reaction took place in a reaction capillary (5.0 m × 0.25 mm) at a temperature of 40 °C. The ABTS•^+^ solution was prepared using 54 mg ABTS•^+^ and 9.4 mg K_2_S_2_O_8_. The chemicals were weighed into a 50 mL volumetric flask and filled up with water. The solution was thoroughly shaken. Afterwards, it was incubated and light-protected at room temperature for about 20 h. Then the solution was diluted with 950 mL water and sonicated for 10 min.

### 2.7. Color

For consumers, the color of honey is one of its most important sensory traits. In the present study, the color was mapped by measuring L* (lightness), a* (degree of greenness/redness), and b* (degree of blueness/yellowness) values in the CIEL*a*b* system [36]. The honey samples were measured using the CM-5 bench-top spectrophotometer (Konica Minolta Holdings K.K, Dietikon, Switzerland).

### 2.8. Statistical Analysis

The data were analyzed using statistical methods and the R 3.5.3 software [37]. A value of 0.05 was required for statistical significance. The normality was assessed with Shapiro–Wilk test. The comparisons of quantitative variables in two groups were conducted with either the Student *t*-test (in the case of normal distribution in both groups), or with Man–Whitney test (otherwise). The correlations between quantitative variables were assessed with either the Pearson’s (in the case of normal distribution of both variables), or with the Spearman’s (otherwise) correlation coefficient. The strength of association was judged with the following scores [38]: (I) |r| ≥ 0.9—very strong, (II) 0.7 ≤ |r| < 0.9—strong, (III) 0.5 ≤ |r| < 0.7—moderate, (IV) 0.3 ≤ |r| < 0.5—weak, (V) |r| < 0.3—very weak.

The clustering was done by the agglomerative hierarchical clustering method (AHC). Typical parameters of the method, e.g., Euclidean distance and Ward connection were used. The variables were standardized prior to the AHC analysis to ensure the same weight for each of them.

The dimension reduction was done with the principal component analysis method (PCA). Two first components were extracted for further analysis.

## 3. Results and Discussion

The phenolic compound profiles, along with total phenolic contents (TPC), total antioxidant activities (AOX), and color parameters (L*, a*, b*) of all samples tested are presented in Table 2.

### 3.1. Phenolic Compound Profiles

In the analyzed honey samples, ten phenolic compounds were detected. The concentration of Chrysin fluctuated from 38 to 108 µg/100 g, whereas caffeic acid varied from 37 to 215 µg/100 g, *p*-coumaric acid from 164 to 788 µg/100 g, and pinocembrin from 27 to 113 µg/100 g. In five cases, the concentration of luteolin was below the detection level and varied between 1 and 105 µg/100 g for the remaining samples. The concentration of quercetin fluctuated from 0.002 to 0.483 mg/100 g, kaempferol from 25 to 161 µg/100 g, galangin from 35 to 154 µg/100 g, trans-ferulic acid from 47 to 315 µg/100 g, and apigenin from 7 to 45 µg/100 g. 

The present study has confirmed earlier findings about honey composition and its link to the floral source, geographical origin, and climatic conditions, not only with the antioxidant activity [15,16,17,18] but also with their characteristic phenolic profile.

However, Jasicka-Misiak et al. [23] identified even more phenolic compounds in Polish honeys than those identified in the present research. Besides the compounds detected in the samples investigated in the present study, also 3-hydroxybenzoic acid, chlorogenic acid, 4-hydroxybenzoic acid, vanillic acid, syringic acid, rosmarinic acid, ellagic acid, and myricetin in heather honey (Calluna vulgaris), 3,4-dihydroxybenzoic, chlorogenic, 4-hydroxybenzoic, 3-hydroxybenzoic, vanillic, ferulic, *p*-coumaric, ellagic, rosmarinic acids as well as myricetin in buckwheat honeys (Fagopyrum esculentum) were found. However, they did not find pinocembrin, luteolin, or apigenin. The levels of phenolic compounds identified in the present study for honeys containing dominant heather and accompanying buckwheat pollens are in agreement with the results provided by Jasicka-Misiak et al. [23]. Mostly, the concentrations determined in the present study are in the lower part of the marked range as described in the literature, with the exception of galangin in heather honeys. However, in sample No. 8, the concentrations were much higher than in the study done by Jasicka-Misiak et al. [23]. Socha et al. [29] also identified a higher number of phenolic compounds in Polish honeys. Besides polyphenols determined in the present study, they also found: Gallic acid, ferulic acid, syringic acid, synaptic acid, and chlorogenic acid, as well as chrysin, hesperetin, and naringenin. The ranges of phenolic compounds identified in both studies were more or less similar. Exceptions concerned lower amounts of chrysin, galangin, kaempferol, and quercetin, and the lack of luteolin and apigenin in the study by Socha et al. [29]. Jasicka-Misiak et al. [39] tested Polish yellow sweet clover (Melilotus officinalis) honeys. There, they also identified different profiles of phenolic compounds: Gallic acid, catechin, 4-hydroxybenzoic acid, caffeic acid, 3-hydroxybenzoic acid, *p*-coumaric acid, ferulic acid, rosmarinic acid, ellagic acid, myricetin, cinnamic acid, quercetin, genistein, pinocembrin, and morin hydrate. The concentrations of polyphenols identified by Jasicka-Misiak [39] were higher than in the present study, with regard to compounds common in both studies.

Moreover, several other research results performed on different varieties all around the world showed qualitative and quantitative differences in the phenolic profiles. Consequently, a phenolic profile is more or less unique. 

Serbian polyfloral honeys contained protocatechuic acid, chlorogenic acid, caffeic acid, *p*-coumaric acid, ellagic acid, rutin, luteolin, quercetin, cis, trans-abscisic acid, apigenin, kaempferol, chrysin, pinocembrin, and galangin. The concentrations of compounds identified in the present study were mostly in the lower part of the ranges, as presented by Gašić et al. [40]. The exceptions were luteolin and quercetin, which showed higher concentrations in the present study. Of note, Gašić et al. [40] did not identify trans-ferulic acid. Habib et al. [41] determined gallic acid, 4-hydroxy-3-methoxybenzoic acid, syringic acid, *p*-coumaric acid, ferulic acid, cinnamic acid, catechin, epicatechin, and rutin in honeys from arid and non-arid regions. They found much lower concentrations of compounds than in the present study. Can et al. [42] detected gallic acid, protocatechuic acid, p-hydroxybenzoic acid, catechin, vanillic acid, caffeic acid, syringic acid, epicatechin, *p*-coumaric acid, ferulic acid, rutin, quercetin, apigenin, kaempferol, and isorhamnetin (only in acacia honey) in Turkish honeys. The concentrations of caffeic and *p*-coumaric acids determined in the present study were in the lower ranges as noted for the Turkish honeys. Similar situation concerned kaempferol. Apart from samples No. 17 and No. 18, the concentrations found in the present study were much higher. Quercetin and apigenin had higher levels in the study described by Can et al. [42]. Pichichero et al. [19] identified gallic acid, chlorogenic acid, *p*-coumaric acid, caffeic acid, myricetin, quercetin, genistein, kaempferol, apigenin, chrysin, and galangin in Italian honeys. Considering the results of the present and the Italian study, the concentrations of kaempferol and caffeic acid were on a similar level, galangin and chrysin were higher in Italian honeys, quercetin generally similar, but in three cases much higher in Polish honeys, whereas the contents of *p*-coumaric acid were mostly higher in Polish honeys. Do Nascimento et al. [43] determined gallic, protocatechuic, cinnamic, and *p*-coumaric acids, as well as quercetin and myricetin in 48 Brazilian honeys. However, it should be noted that protocatechuic was detected only in two, myricetin in three, cinnamic acid in four, and *p*-coumaric in eight cases. When comparing the results obtained in Brazil with the outcomes of the present study, it can be noted that *p*-coumaric acid concentrations were higher in Polish honeys, whereas quercetin contents were generally on similar level. Chinese honeys characterized by Zhao et al. [44] contained gallic acid, protocatechuic acid, *p*-hydroxybenzoic acid, caffeic acid, *p*-coumaric acid, rutin, rosmarinic acid, quercetin, and kaempferol. The concentrations of caffeic acid and kaempferol were much higher than in the case of Polish honeys of the present study. The determined levels of *p*-coumaric acid in Polish honeys were in the lower range of the concentrations identified for Chinese honeys. Generally, Chinese honeys also contained more quercetin. There were, however, tree exceptions (samples Nos. 1, 2, and 22), where the concentrations were similar to those noted by Zhao et al. [44]. Oroian and Ropciuc [45] identified apigenin, caffeic acid, chrysin, galangin, gallic acid, isorhamnetin, kaempherol, luteolin, myricetin, *p*-coumaric acid, pinocembrin, and quercetin in Romanian honeys. Mostly higher concentrations of the detected phenolic compounds than in the case of Polish honeys were suggested, but the form of the results presented made it impossible to make a direct comparison. In Nordic honeys, Salonen et al. [46] determined more diverse phenolic profiles than in the present study. They identified 33 phenolic compounds: 14 cinnamic acid derivatives, 6 phenolic acids, and 13 flavonoids. 

### 3.2. Total Phenolic Content (TPC)

Total phenolic content varied between 3.43 mg GAE/100 g for rapeseed honey (sample No. 17) and 22.33 mg GAE/100 g for multifloral honey, containing buckwheat accompanying pollen (sample No. 11). 

Similarly, for Polish honeys, Socha et al. [29] noted the TPC ranging from 4.46 (rapeseed honey) to 15.04 mg GAE/100 g (buckwheat honey). Moreover, Wesołowska and Dżugan [22] determined the TPC in a similar range, i.e., from 8.48 mg GAE/100 g (for linden honey) to 28.15 mg GAE/100 g (for buckwheat honey) for honeys of Polish origin. Nevertheless, generally higher TPC for Polish honeys was determined, ranging from 69.4 mg GAE/100 g for willow honey to 160.7 mg GAE/100 g for buckwheat honey [47], from 14.28 mg GAE/100 g for black locust honey to 111.3 mg GAE/100 g for buckwheat honey [48], from 17.57 mg GAE/100 g for rapeseed honey to 189.52 mg GAE/100 g for heather honey [28], as well as from 67.55 mg GAE/100 g for yellow sweet clover honeys [38], or from 59.9 mg GAE/100 g for heather honey to 121.4 mg GAE/100 g for buckwheat honey [23].

Accordingly, the results of research conducted in different parts of the world indicate a high variation of TPC and show that the results obtained in the present study are mostly in lower ranges. Romanian heather honeys tested by Moise et al. [49] contained, on average, 54.22 mg GAE/100 g of phenolic compounds. Pérez Martín et al. [50] noted the average TPC of 102 mg GAE/100 g for Spanish honeydew honeys and 45 mg GAE/100 g for nectar Spanish honeys. Bertoncelj et al. [51] for Slovenian honeys measured total phenol content from 44.8 (acacia honey) to 241.4 mg GAE/kg (for honeydew honey). The TPC of Brazilian eucalyptus honeys and wild honeys examined by Bueno-Costa et al. [51] fluctuated from 61.16 to 111.37 mg GAE/100 g. Moreover, Do Nascimento et al. [43] determined TPC in the range of 26.0 to 100.0 mg GAE/100 g for Brazilian honeys [52]. Similar values were detected for Sicilian monofloral honeys with 16.5 to 133.3 mg GAE/100 g [53], for Tunisian honeys (32.17 to 119.42 mg GAE/100 g [54], Can et al. [42] for Turkish honeys (16.02 to 120.04 mg GAE/100 g), Habib et al. [41] (30.81 to 132.6 mg GAE/100 g), or Flanjak et al. [8] for Croatian honey (39.1 to 318.6 mg GAE/100 g). Alvarez-Suarez et al. [55], on the other hand, detected an average value of 54.30 mg GAE/100 g for polyfloral honeys from Cuba. Serem and Bester [56] noted TPC ranging from 68.85 to 167.96 mg GAE/100 g for southern African honeys. Spanish honeys studied by Escuredo et al. [57] contained, on average, 112.8 mg GAE/100 g, with the lowest value of 78.4 mg GAE/100 g for eucalyptus honey and the highest of 181 mg GAE/100 g for heather honey. Honeydew honeys studied by Flores, Escuredo and Seijo [31] contained, on average, 100 to 154.4 mg GAE/100 g polyphenols, depending on the production year. Gašić et al. [40] determined the TPC ranging from 6 to 136 mg GAE/kg for Serbian polyfloral honeys. The TPC of Chinese honeys ranged from 33.3 to 50.2 mg GAE/100 g [44] and from 13.5 to 63.9 mg GAE/100 g [58]. A wide range of TPC was noted by Noor et al. [59] for Pakistani honeys (1.33 to 252 mg GAE/100 g). The TPC of Palestinian honeys ranged from 26.9 to 70.7 mg GAE/100 g [60]. Nevertheless, there are studies showing similar TPC ranges as obtained in the present study. Czech honeys had the TPC ranging from 8.36 mg GAE/100 g for linden honey to 24.25 mg GAE/100 g for honeydew honey [61], Nordic honeys from 0.94 mg GAE/100 g for willow honey to 5.52 mg GAE/100 g for heather honey [46] and Italian honeys from 6.05 mg GAE/100 g (Sulla honey) to 27.6 mg GAE/100 g (honeydew honey) [19].

### 3.3. Total Antioxidant Activity (AOX) 

The antioxidant activity of the honey samples tested varied between 41.42 mg GAE/100 g for rapeseed honey (sample No. 21) and 83.16 mg GAE/100 g also for rapeseed honey (sample No. 16).

By comparison, Czech honeys studied by Lachman et al. [61] showed generally lower antioxidant activity. Those values ranged from 9.87 to 44.2 mg GAE/kg. It is, however, difficult to compare values with the literature because of different presentation of the results (e.g., expressed as percentage inhibition of DPPH or further radicals). Nevertheless, the results obtained by other authors show high variability in honeys [15,16,17,18], indicating that the floral source, geographical origin, and ecophysiological conditions affect the antioxidant properties of honey. For instance, Bertoncelj et al. [51] noted DPPH-IC_50_ values from 8.1 to 13.9 mg/mL for Slovenian honeys, Zhao et al. [44] 87.5 to 136.2 mg/mL for Chinese honeys, Al-Farsi et al. [62] 7.8 to 72.3 mg/mL for Omani honeys, Flanjak et al. [8] 8.69 to 125.48 mg/mL for Croatian honeys, Escuredo et al. [57] 8.6 and 17.8 mg/mL for Spanish honeys. Sicilian monofloral honeys as characterized by Attanzio et al. [53] showed antioxidant activity (DPPH assay) ranging from 8.5 to 238.4 μmol TE/100 g, whereas honeys from Southern Africa ranged from 42 to 372 μmol TE/100 g [56]. The average antioxidant activity (expressed as DPPH radical scavenging activity) of Romanian heather honeys was 56% [49], whereas for Spanish honeydew and nectar honeys 60.8% and 34.8% were determined accordingly [50].

A high variability of radical scavenging activity in DPPH assay results can also be noticed in Polish honeys: Wesołowska and Dżugan [22] noted an average value of 12.4% for linden honey, 13.6% for rapeseed honey, and 48.6% for buckwheat honey, whereas Socha et al. [29] reported 34.5%, 18.2%, and 46.4% accordingly, and Wilczyńska [28] 100% for buckwheat honey. However, the results for Polish honeys show the tendency of higher radical scavenging activity with regard to darker colored honeys [22,28,29,48].

When evaluating the contribution of colored compounds to antioxidant activity, two fractions have to be kept in mind. Melanins that are formed from the oxidation (‘browning’) of polyphenols, usually catalyzed by (plant) endogenous enzymes [63], but also non-enzymatic browning, often described as the Maillard reaction. The latter is occurring in honey as well, as the hive has a certain temperature, leading to reactions of sugars and amino compounds (e.g., proteins, peptides, amino acids), ending up in the formation of melanoidins. Gaining additional complexity, Brudzynski and Miotto [64] showed for honey that the heat treatment causes increased incorporation of phenolics during the formation of high molecular weight melanoidins.

Melanins, as well as melanoidins, are very good antioxidants [65,66,67]. Resulting from the chemical structure, the polymerization of phenolic compounds provides numerous structural features (e.g., aromatic rings, double bonds, hydroxyl groups) for scavenging radicals. Almost similar structures can be found in melanoidins, which result from the well-known Maillard reaction [66,67]. Moreover, two types of covalent protein-polyphenol-reaction products have been described in honey. In that case, oligomerization of phenolic monomers occurred prior to protein binding [68].

### 3.4. Color Parameters (L*, a*, b*)

The color of honey depends on the floral source and mineral content and usually varies between water white to dark amber [69]. In the present study, L* color parameter ranged from 23.18 (for honeydew honey) to 59.91 (for multifloral honey with significant amounts of linden and white/sweet clover pollens), whereas b* parameter ranged from −1.61 (for multifloral honey with significant amounts of linden and white/sweet clover pollens) to 11.6 (for multifloral honey with significant amounts of white/sweet clover, phacelia and buckwheat pollens). An a* parameter ranged from 7.59 (for rapeseed honey) to 31.33 (for multifloral honey with significant amounts of white/sweet clover, phacelia and buckwheat pollens).

In general, the color values determined were in a similar range, as reported in the literature [8,48]. For Croatian honeys, Flanjak et al. [8] noted average L* values ranging from 29.07 (for honeydew honey) to 46.15 (for black locust honey), a* values from −1.08 (for black locust honey) to 7.95 (for chestnut honey), and b* values from 4.79 (for honeydew honey) to 19.62 (for lime honey). For Polish honeys, Kuś et al. [48] determined L* values ranging from 41.5 for (buckwheat honey) to 86.2 for (black locus honey), a* values from −1.6 (for black locust honey) to 31.9 (for buckwheat honey), and b* values from 19.6 (for black locust honey) to 69.2 (for buckwheat honey). Comparable values for L* and a* were also noted by Bertoncelj et al. [51] for Slovenian mulitfloral honeys. There, L* values ranged from 50.30 to 57.30 (53.87 on average), whereas a* values were from −0.90 to 5.77 (2.25 on average), and b* values between 43.10 and 49.22 (46.45 on average).

### 3.5. Comparison of Organic and Conventional Samples

In order to compare organic and conventional honey samples, the PCA method was applied. The first two main components explained 49.71% of the total variance, with the first component (PC1) 28.18% and the second one (PC2) 21.53%.

**Figure 1:**
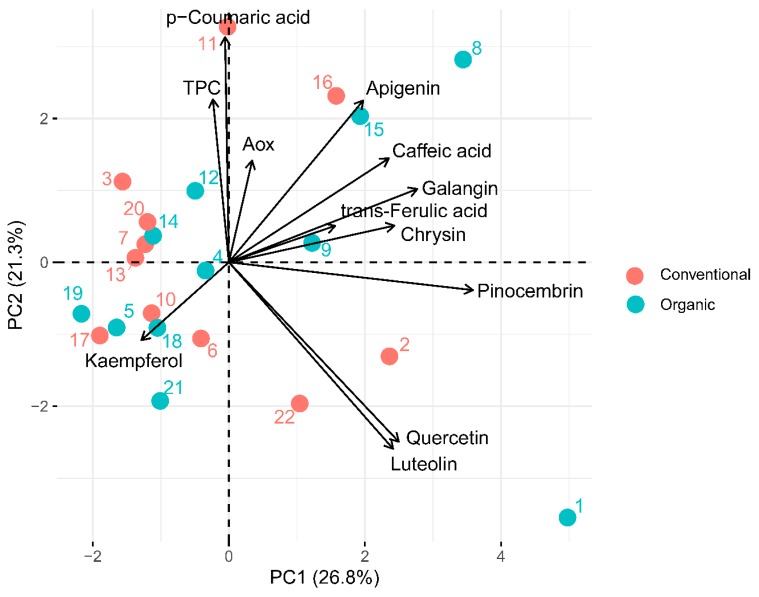
The results of the principal component analysis (PCA) with ● organic and ● conventional honey.

Based on the biplot (Figure 1), it can be noticed that the first component indicates high concentrations of apigenin, caffeic acid, galangin, chrysin, pinocembrin, quercetin, and luteolin, but low concentrations of kaempferol in the products. The second component indicates a high TPC and a high content of *p*-coumaric acid and apigenin with low concentrations of quercetin and luteolin.

Moreover, Figure 1 shows that straightforward discrimination of organic and conventional samples is not possible. The results of the analysis show that most of the samples tested had low concentrations of the first component. The other samples (sample Nos. 1, 2, and 22) were characterized by high concentrations of pinocembrin, quercetin, and luteolin, whereas samples Nos. 8, 9, 15, and 16 by high concentrations of apigenin, caffeic acid, galangin, and chrysin.

On the other hand, the comparison of the average concentrations of the single polyphenols, AOX, TPC, and L* a* b* color parameters proved that organic honeys contain significantly more chrysin than their corresponding conventional counterparts (Table 3).

### 3.6. Correlations between Tested Parameters of Honey

As only the average content of chrysin seems to make the differentiation of organic and conventional honeys possible, correlations between pollen content and phenolic profile along with AOX, TPC, and color parameters (L*, a*, B*) were tested as well.

In total, 47 statistically significant correlations were detected (Table 4). There were strong, positive correlations with regard to buckwheat pollen and three further parameters (TPC, a*, b*). In this way, it was confirmed that buckwheat pollen strongly adds red and yellow tones to the color of honey. It also moderately negatively affects the lightness of honey. On the other hand, plum/apple trees and chervil pollens have a positive influence on the lightness of honey. Moreover, buckwheat honeys scored the highest AOX among all samples tested. However, the high impact of buckwheat pollen on TPC was not reflected in its correlation with AOX. Buckwheat pollen only moderately correlated with *p*-coumaric acid, which is further, according to Socha et al. [29], weakly correlated with the antioxidant activity of honey. In contrast to buckwheat pollen, chervil pollen affected the TPC moderately negatively. The concentrations of selected polyphenols were correlated with specific pollens in the present study, as well: Pinocembrin showed moderate, positive correlation with white/sweet clover pollen, kaempferol with rapeseed pollen, and luteolin with chervil. However, kaempferol was moderately negatively correlated with buckwheat pollen and *p*-coumaric acid with willow pollen.

Besides existing interdependencies between color parameters, the TPC was also correlated with them. In the case of b* values, the correlation was strong and positive, in the case of a* values moderate and positive, whereas in the case of L* values moderate and negative. This means that the higher the TPC, the more yellow and red color tones honey has. At the same time, honey is darker. Similar results were noted by Bertoncelj et al. [51], Kuś et al. [48], and Kavanagh et al. [15]. In the present study, the light color of honey was also moderately negatively correlated with AOX and *p*-coumaric acid. As mentioned above, other studies also showed that a higher antioxidant activity can be found in the case of a darker color of the honey samples, resulting from higher concentrations of melanoidins, being comparatively good antioxidants [59].

The correlations between selected phenolic compounds were determined, as well. A particularly interesting finding was the existence of strong dependencies between the concentrations of pinocembrin and galangin. Of note, these two compounds are reported to ameliorate insulin resistance [70]. Moderate correlations with regard to the pairs luteolin and quercetin, chrysin and pinocembrin, caffeic acid and pinocembrin, chrysin and apigenin, as well as chrysin and galangin were observed. These dependencies may indicate that some polyphenols can be expected to occur together in honey.

Although some authors indicated that the phenolic compounds in honey significantly contribute to its antioxidant activity and capacity, e.g., [8,12,19,20,21,29,48,71], in the present study an only moderate positive correlation between AOX and TPC was determined. This suggests that the antioxidant activity of honey is diversified and as stated by Gheldof et al. [3,72] does not solely result from phenolic compounds, but seems to be a consequence of the combined activity of phenolic compounds, melanins thereof, peptides, organic acids, enzymes, and Maillard reaction products.

In this way, the earlier mentioned lack of correlation between AOX and buckwheat pollen, despite a strong interdependence between the TPC and buckwheat pollen, can be explained. Buckwheat pollen seems to be associated with high amounts of polyphenol compounds of weak or moderate antioxidant activity. A positive dependence between the antioxidant activity of honey, determined by the oxygen radical absorbance capacity (ORAC) assay, and the TPC was also noted by Gheldof and Engeseth [73]. Nevertheless, in their research, the correlation was strong (*R^2^* = 0.9497). However, as stated by Küçük et al. [74] and Gül and Pehlivan [75], the mismatch between the content of phenolic substances and the antioxidant activity can be explained by the varying radical scavenging activities of the phenolic compounds compiled in the varying profiles.

## 4. Conclusions

The findings of the present study contribute to the discussion on the health benefits of organic farming by indicating significant differences concerning chrysin as a marker compound.

Moreover, the elaborated phenolic profiles, which include pollen contents, vastly contribute to increasing knowledge about Polish honeys. The PCA results have shown that most of the samples had a high content of apigenin, caffeic acid, galangin, chrysin, pinocembrin, quercetin, and luteolin, while they were low in kaempferol. However, the concentrations were mostly in the lower part of the ranges, as described in other studies. Moreover, the TPC values were within the ranges of Polish honeys, already described in the literature. Total antioxidant activity analysis results are also in line with earlier studies, indicating that darker colored honeys in comparison to the light-colored ones, have a higher AOX. The determined values of the L*a*b* color parameters were in similar ranges to those reported in the literature.

Furthermore, the present study not only confirmed the link between TPC and color (the higher TPC, the more yellow and red color tones can be observed in honey) but also indicated positive influences on the lightness of honey resulting from plum/apple tree and chervil pollens. The light color of honey was moderately, negatively correlated with AOX and *p*-coumaric acid content.

Despite the fact that some authors pointed out that the phenolic compounds in honey significantly contribute to its antioxidant activity, in the present study, only a moderate positive correlation between AOX and TPC was found.

An additional value of this research is that it has indicated the correlation of pinocembrin and galangin, two very interesting compounds in honey that are reported to ameliorate insulin resistance.

## Figures and Tables

**Table 1:** Research material with its specific characteristics.

Sample No	Variety-Declaration by Producer	The Determined Pollens of Nectar Honeys/the Determined Conductivity of Honeydew Honeys					Variety According to Pollen Analysis Results
		Dominant Pollens >45%/Conductivity	Accompanying Pollens <15–45%	Important Pollens <3–15%	Other Pollens <3%	Wind Pollinating and no Nectar Producing Plants Pollens	
1	Linden *		linden: 22.7% white/sweet clover: 19.4%	Brassicaceae: 12.9% chervil: 12.4% plum/apple tree: 7.1% willow: 7.0% red clover: 4.8% thistle: 3.2%	dandelion: 2.2% blue cornflower: 1.6% other: 6.7%	oak: 2.1% sorrel: 1.5% *Plantago lanceolata* L.: 2.0%	Multifloral
2	Linden **		Brassicaceae: 42.3%	white/sweet clover: 12.7% chervil: 8.5% plum/apple tree: 7.4% linden: 6.3% blue cornflower: 5.8% red clover: 3.2%	dandelion: 2.6% vicia: 2.5% thistle: 2.1% phacelia: 1.1% other: 5.5%	oak: 5.8% sorrel: 2.4%	Multifloral
4	Goldenrod *		Brassicaceae: 43.4% goldenrod: 25.5%	plum/apple tree: 11.1% heather: 4.8% white/sweet clover: 3.4%	phacelia: 2.7% red clover: 2.1% linden: 0.7% other: 5.5%	goosefoot: 0.7% corn: 0.5% pine: 0.3%	
3	Goldenrod **	goldenrod: 55.8%	phacelia: 21.5%	heather: 7.7% buckwheat: 6.1% Brassicaceae: 5.5%	blue cornflower: 1.1% other: 2.3%	mugwort: 1.1% sorrel: 0.6% *Plantago lanceolata* L.: 0.5%	Goldenrod
5	Dandelion*	rapeseed: 79.7%		willow: 11.1% plum/apple tree: 6.1%	other: 3.1%	oak: 0.9%	Rapeseed
6	Dandelion **	rapeseed: 53.4%	willow: 27.7%	raspberry: 6.5% plum/apple tree: 5.9% chervil: 5.6%	other: 0.9%	*Plantago lanceolata* L.: 3.3%	Rapeseed
8	Heather *	heather: 65.6%		phacelia: 10.0% buckwheat: 9.7% rapeseed: 4.3% blue cornflower: 3.8%	*Achillea millefolium* L.: 2.2% linden: 0.9% other: 5.4%	sorrel: 1.2%	Heather
7	Heather **	heather: 47.9%		phacelia: 12.7% rapeseed: 11.9% blue cornflower: 7.3% white/sweet clover: 5.7% red clover: 4.6% buckwheat: 4.5%	other: 5.4%		Heather
9	Acacia *		rapeseed: 41.2% raspberry: 21.8%	maple: 10.1% *Frangula alnus* Mill.: 8.4% willow: 7.6% blue cornflower: 5.1%	other: 5.8%		Multifloral
10	Acacia **	rapeseed: 50.1%	phacelia: 37.4%	plum/apple tree: 3.7%	*Achillea millefolium* L.: 2.6% sunflower: 2.1% blue cornflower: 1.7% vicia: 1.1%		Rapeseed
12	Buckwheat *	rapeseed: 45.5%	buckwheat: 19.9%	sunflower: 10.9% white/sweet clover: 9.6% vicia: 3.8% thistle: 3.2%	other: 7.1%	mugwort: 5.8% corn: 1.7% *Plantago lanceolata* L.: 0.6%	Rapeseed
11	Buckwheat **		white/sweet clover: 39.7% phacelia: 19.6% buckwheat: 17.2%	blue cornflower: 9.5% red clover: 7.1%	other: 6.9%	pine: 0.9% oak: 0.8%	Multifloral
14	Honeydew *		Conductivity: 1.05 mS/cm		raspberry/blueberry, white clover, *Plantago lanceolata* L., Frangula alnus Mill., blue cornflower.		Coniferous honeydew
13	Honeydew **		Conductivity: 0.67 mS/cm		raspberry/blueberry, *Impatiens* L., linden, *Heracleum* L., linden, grass, dandelion, *Achillea millefolium* L., red clover.		Nectar-honeydew
15	Bean *		rapeseed: 44.6% white/sweet clover: 17.1%	raspberry: 12.7% Asteraceae: 10.6% thistle: 4.2%	phacelia: 2.1% other: 8.7%	mugwort: 62.2% corn: 0.8% *Plantago lanceolata* L.: 1.5%	Multifloral
16	Bean **	rapeseed: 59.8%		*Medicago sativa* L.: 10.7% raspberry: 8.1% white/sweet clover: 6.3% blue cornflower: 3.6%	chervil: 2.7% vicia: 2.6% thistle: 1.8% other: 4.4%		Rapeseed
18	Rapeseed *	rapeseed: 86.4%		willow: 6.8% maple: 3.1%	plum/apple tree: 2.1% other: 1.6%		Rapeseed
17	Rapeseed **	rapeseed: 86.2%		willow: 7.7% plum/apple tree: 4.5%	other: 1.6%	pine: 2.1%	Rapeseed
19	Wild raspberry *	rapeseed: 79.6%		phacelia: 13.4%	willow: 2.2%; blue cornflower: 2.1% red clover: 0.9% other: 1.8%	pine: 3.9%	Rapeseed
20	Wild raspberry **	rapeseed: 54.6%	raspberry: 26.6%	plum/apple tree: 8.5% phacelia: 4.1%	buckwheat: 1.5% dandelion: 1.1% Asteraceae: 0.7% other: 2.9%	oak: 0.7%	Rapeseed
21	Forest *	rapeseed: 56.1%	willow: 15.2%	*Frangula alnus* Mill.: 11.7% raspberry: 8.4% white/sweet clover: 3.6%	red clover: 0.8% other: 4.2%	oak: 2.6% *Plantago lanceolata* L.: 3.5% sorrel: 1.7% goosefoot: 1.4%	Rapeseed
22	Forest **		rapeseed: 38.9%	willow: 14.6% chervil: 13.7% maple: 8.9% linden: 7.3% white/sweet clover: 6.6% raspberry: 4.3%	*Frangula alnus* Mill.: 1.9% sunflower: 0.6% Other: 3.2%	oak: 0.9% sorrel: 0.3%	Multifloral

*—organic sample; **—conventional sample.

**Table 2:** Phenolic compound profile, total phenolic content (TPC), total antioxidant activity (AOX), and color parameters (L*, a*, b*) of some selected honey samples.

Sample No	1 *	2 **	3 **	4 *	5 *	6 **	7 **	8 *	9 *	10 **	11 **	12 *	13 **	14 *	15 *	16 **	17 **	18 *	19 *	20 **	21 *	22 **
Parameter $\bar{x}\;\left(SD\right)$																						
AOX [mg GAE/100 g]	53.33 (2.36)	71.58 (10.03)	77.58 (2.42)	53.94 (0.90)	54.42 (2.04)	45.65 (2.54)	56.25 (8.73)	47.63 (4.08)	46.23 (12.47)	55.12 (2.02)	67.28 (4.14)	58.30 (0.18)	45.58 (0.20)	50.24 (0.65)	51.81 (1.77)	83.16 (2.56)	53.37 (3.43)	54.37 (1.95)	54.66 (1.03)	52.90 (7.45)	41.42 (1.50)	55.75 (0.97)
TPC [mg GAE/100 g]	3.96 (0.49)	3.86 (0.38)	8.15 (0.49)	5.81 (0.50)	5.26 (0.64)	4.08 (0.22)	14.08 (1.66)	12.40 (0.77)	4.15 (0.07)	4.15 (0.04)	22.33 (0.81)	17.95 (1.57)	6.79 (0.76)	7.24 (0.24)	4.07 (0.14)	3.73 (0.26)	3.43 (0.19)	3.93 (0.21)	4.21 (0.48)	6.38 (0.07)	5.69 (0.45)	4.05 (0.02)
Chrysin [µg/100 g]	86 (46)	79 (23)	54 (10)	101 (5)	85 (11)	89 (18)	69 (8)	108 (7)	79 (7)	66 (2)	67 (4)	79 (3)	60 (11)	71 (3)	89 (14)	79 (5)	53 (6)	66 (3)	59 (11)	38 (6)	58 (5)	74 (5)
Caffeic acid [µg/100 g]	120 (16)	98 (0)	70 (3)	46 (0)	56 (0)	54 (2)	51 (9)	194 (24)	215 (68)	184 (75)	110 (38)	72 (6)	94 (17)	37 (6)	203 (28)	157 (18)	52 (3)	61 (4)	42 (0)	107 (20)	76 (2)	97 (2)
*p*-Coumaric acid [µg/100 g]	181 (54)	291 (15)	526 (35)	327 (21)	338 (14)	166 (157)	266 (125)	407 (8)	272 (13)	164 (6)	788 (48)	427 (7)	473 (43)	423 (16)	539 (55)	510 (65)	288 (10)	210 (13)	276 (9)	534 (26)	170 (10)	310 (17)
Pinocembrin [µg/100 g]	113 (32)	98 (6)	30 (3)	49 (2)	29 (2)	51 (14)	40 (2)	76 (3)	69 (16)	38 (3)	71 (6)	59 (1)	27 (23)	35 (4)	63 (9)	59 (4)	41 (5)	48 (4)	33 (3)	35 (6)	42 (10)	58 (6)
Luteolin [µg/100 g]	105 (24)	56 (6)	nd	nd	19 (1)	9 (1)	8 (0)	11 (2)	4 (2)	nd	3 (1)	7 (1)	10 (3)	9 (0)	4 (1)	2 (0)	nd	1 (0)	nd	5 (3)	45 (1)	64 (10)
Quercetin [µg/100 g]	483 (131)	248 (53)	35 (4)	36 (2)	42 (2)	37 (4)	31 (3)	19 (3)	25 (1)	22 (4)	27 (0)	57 (4)	49 (1)	7 (1)	2 (8)	11 (0)	43 (1)	20 (14)	10 (4)	33 (18)	63 (2)	233 (35)
Kaempferol [µg/100 g]	36 (19)	99 (12)	51 (6)	76 (5)	146 (4)	117 (44)	80 (34)	68 (7)	80 (5)	105 (5)	35 (2)	40 (1)	92 (38)	25 (2)	88 (10)	71 (9)	161 (11)	158 (14)	99 (2)	54 (7)	63 (2)	63 (10)
Galangin [mg/100 g]	126 (89)	64 (16)	40 (9)	63 (0)	35 (0)	91 (14)	63 (15)	154 (34)	118 (11)	58 (5)	70 (3)	68 (2)	51 (10)	58 (2)	107 (9)	143 (10)	86 (10)	117 (9)	54 (5)	61 (8)	37 (2)	63 (5)
trans-Ferulic acid [µg/100 g]	252 (66)	184 (20)	229 (13)	170 (15)	79 (102)	70 (37)	81 (33)	167 (7)	128 (17)	84 (4)	115 (14)	47 (4)	204 (115)	221 (12)	283 (28)	213 (32)	106 (9)	112 (7)	87 (1)	315 (15)	128 (6)	179 (4)
Apigenin [µg/100 g]	7 (2)	22 (7)	10 (1)	12 (1)	13 (0)	17 (3)	18 (3)	45 (1)	15 (0)	7 (0)	15 (2)	11 (1)	13 (1)	11 (0)	29 (3)	23 (2)	10 (1)	13 (1)	11 (0)	8 (1)	11 (0)	9 (0)
L *	59.91 (0.42)	59.07 (0.10)	29.26 (0.53)	52.83 (0.67)	41.06 (1.05)	52.46 (0.74)	29.27 (0.08)	25.58 (0.26)	36.42 (0.68)	55.83 (0.46)	27.71 (0.45)	36.04 (0.68)	33.32 (0.97)	23.18 (1.24)	47.48 (0.35)	34.38 (0.84)	54.55 (0.36)	52.47 (0.35)	43.82 (1.25)	38.13 (1.73)	35.26 (0.54)	57.91 (0.68)
a *	−1.61 (0.12)	−1.23 (0.19)	1.89 (0.48)	−0.39 (0.09)	0.38 (0.11)	−0.91 (0.04)	7.40 (0.17)	5.30 (0.27)	−1.38 (0.07)	−1.12 (0.00)	11.60 (0.68)	10.14 (0.42)	6.04 (0.16)	5.02 (0.16)	−1.03 (0.00)	−1.00 (0.00)	−1.17 (0.01)	−1.02 (0.05)	−1.17 (0.09)	1.53 (0.26)	0.98 (0.02)	−0.84 (0.17)
b *	11.49 (0.91)	13.07 (0.33)	21.61 (0.78)	16.01 (0.09)	20.19 (0.16)	15.52 (0.07)	29.06 (0.78)	24.32 (0.44)	15.97 (0.56)	12.86 (0.26)	31.33 (1.41)	30.45 (1.30)	27.51 (0.54)	23.55 (0.62)	9.96 (0.17)	7.59 (0.30)	13.35 (4.87)	13.71 (0.31)	13.46 (0.28)	22.10 (0.37)	20.01 (0.34)	15.36 (0.12)

*SD*—standard deviation; nd—not detected; *—organic sample, **—conventional sample.

**Table 3:** Phenolic profiles, total phenols content (TPC), total antioxidant activity (AOX), and color parameters (L*, a*, b*) of organic and conventional honeys of Polish origin.

Parameter	Conventional (N = 11) $\bar{x}\;\left(SD\right)$	Organic (N = 11) $\bar{x}\;\left(SD\right)$	*p* *
Chrysin [mg/100 g]	0.066 (0.014)	0.08 (0.016)	0.045 (P)
Caffeic acid [mg/100 g]	0.098 (0.043)	0.102 (0.069)	0.797 (NP)
*p*-Coumaric acid [mg/100 g]	0.392 (0.19)	0.325 (0.117)	0.326 (P)
Pinocembrin [mg/100 g]	0.05 (0.021)	0.056 (0.024)	0.529 (P)
Luteolin [mg/100 g]	0.014 (0.023)	0.018 (0.032)	0.817 (NP)
Quercetin [mg/100 g]	0.068 (0.086)	0.069 (0.139)	0.511 (NP)
Kaempferol [mg/100 g]	0.084 (0.036)	0.08 (0.042)	0.792 (P)
Galangin [mg/100 g]	0.072 (0.028)	0.085 (0.040)	0.669 (NP)
trans-Ferulic acid [mg/100 g]	0.162 (0.077)	0.152 (0.075)	0.769 (P)
Apigenin [mg/100 g]	0.014 (0.006)	0.016 (0.011)	0.792 (NP)
AOX [mg GAE/100 g]	60.38 (12.57)	51.49 (5.44)	0.054 (P)
TPC [mg GAE/100 g]	7.37 (5.61)	6.79 (4.27)	0.748 (NP)
L *	42.899 (12.926)	41.277 (11.455)	0.898 (NP)
a *	2.017 (4.400)	1.383 (3.800)	0.606 (NP)
b *	19.032 (7.761)	18.101 (6.234)	0.760 (P)

*p = normality in both groups, Student *t*-test; NP = lack of normality in at least one group, Mann–Whitney test.

**Table 4:** Correlations between the parameters tested.

Variables		Correlation Coefficient		*p* * Pearson’s Correlation Coefficient	Dependency Direction	Dependence Strength
Buckwheat pollen	TPC		0.755	*p* < 0.001 NP	positive	strong
Buckwheat pollen	a *		0.708	*p* < 0.001 NP	positive	strong
Buckwheat pollen	b *		0.705	*p* < 0.001 NP	positive	strong
White/sweet clover pollen	pinocembrin		0.659	*p* = 0.001 NP	positive	moderate
Plum/apple tree pollen	L *		0.653	*p* = 0.001 NP	positive	moderate
Willow pollen	*p*-coumaric acid		−0.591	*p* = 0.004 NP	negative	moderate
Rapeseed pollen	kaempferol		0.574	*p* = 0.005 NP	positive	moderate
Buckwheat pollen	L *		−0.567	*p* = 0.006 NP	negative	moderate
Chervil pollen	TPC		−0.559	*p* = 0.007 NP	negative	moderate
Chervil pollen	luteolin		0.522	*p* = 0.013 NP	positive	moderate
Chervil pollen	L *		0.516	*p* = 0.014 NP	positive	moderate
Buckwheat pollen	kaempferol		−0.51	*p* = 0.015 NP	negative	moderate
Chervil pollen	b *		−0.485	*p* = 0.022 NP	negative	weak
Chervil pollen	pinocembrin		0.483	*p* = 0.023 NP	positive	weak
Heather pollen	TPC		0.476	*p* = 0.025 NP	positive	weak
Buckwheat pollen	*p*-coumaric acid		0.46	*p* = 0.031 NP	positive	weak
Willow pollen	TPC		−0.455	*p* = 0.033 NP	negative	weak
Chervil pollen	quercetin		0.447	*p* = 0.037 NP	positive	weak
Brassicaceae pollen	quercetin		0.445	*p* = 0.038 NP	positive	weak
Rapeseed pollen	trans-ferulic acid		−0.44	*p* = 0.04 NP	negative	weak
Raspberry pollen	caffeic acid		0.439	*p* = 0.041 NP	positive	weak
Phacelia pollen	TPC		0.43	*p* = 0.046 NP	positive	weak
a *	b *		0.888	*p* < 0.001 NP	positive	strong
Pinocembrin	galangin		0.801	*p* < 0.001 NP	positive	strong
TPC	b *		0.8	*p* < 0.001 NP	positive	strong
L *	a *		−0.758	*p* < 0.001 NP	negative	strong
L *	b *		−0.695	*p* < 0.001 P	negative	moderate
TPC	a *		0.661	*p* = 0.001 NP	positive	moderate
TPC	L *		−0.651	*p* = 0.001 NP	negative	moderate
Luteolin	quercetin		0.649	*p* = 0.001 NP	positive	moderate
Chrysin	pinocembrin		0.596	*p* = 0.003 NP	positive	moderate
Caffeic acid	pinocembrin		0.568	*p* = 0.006 NP	positive	moderate
*p*-Coumaric acid	L *		−0.564	*p* = 0.006 P	negative	moderate
AOX	L *		−0.542	*p* = 0.009 NP	negative	moderate
AOX	TPC		0.541	*p* = 0.009 NP	positive	moderate
Chrysin	apigenin		0.527	*p* = 0.012 NP	positive	moderate
Chrysin	galangin		0.514	*p* = 0.014 NP	positive	moderate
Aox	b *		0.505	*p* = 0.017 NP	positive	moderate
*p*-Coumaric acid	a *		0.496	*p* = 0.02 NP	positive	weak
Caffeic acid	galangin		0.48	*p* = 0.024 NP	positive	weak
Kaempferol	a *		−0.47	*p* = 0.027 NP	negative	weak
Kaempferol	L *		0.465	*p* = 0.029 P	positive	weak
*p*-Coumaric acid	kaempferol		−0.439	*p* = 0.041 P	negative	weak
Quercetin	AOX		−0.437	*p* = 0.042 NP	negative	weak
*p*-Coumaric acid	b *		0.432	*p* = 0.045 P	positive	weak
Galangin	apigenin		0.432	*p* = 0.045 NP	positive	weak
Kaempferol	trans-ferulic acid		−0.423	*p* = 0.05 P	negative	weak

p* = normality of both parameters Pearson’s correlation coefficient; NP = lack of normality of at least one parameter Spearman’s correlation coefficient.
